# 1072. SARS-CoV-2 Antibody Levels Associate with Neutrophil Activation

**DOI:** 10.1093/ofid/ofac492.913

**Published:** 2022-12-15

**Authors:** Seth Warner, Rui Miao, Marcos J Ramos-Benitez, Xin Tian, Robert Reger, Peter D Burbelo, Yogendra Kanthi, Yogendra Kanthi, Jeffrey I Cohen, Anthony F Suffredini, Steven D Nathan, Richard W Childs, Richard W Childs, Richard W Childs, Daniel S Chertow, Jeffrey R Strich

**Affiliations:** Critical Care Medicine Department, National Institutes of Health, Bethesda, Maryland; Office of Biostatistics Research, National Heart, Lung, and Blood Institute, NIH, Bethesda, Maryland; Critical Care Medicine Department, National Institutes of Health Clinical Center, Bethesda, MD, USA. Postdoctoral Research Associate Training Program, National Institute of General Medical Sciences, Bethesda, MD, USA, Bethesda, Maryland; Office of Biostatistics Research, National Heart, Lung, and Blood Institute, NIH, Bethesda, MD, USA, Bethesda, Maryland; Laboratory of Transplantation Immunotherapy, National Heart, Lung, and Blood Institute, NIH, Bethesda, MD, USA, Bethesda, Maryland; National Institute of Dental and Craniofacial Research, National Institutes of Health, Bethesda, MD, USA, Bethesda, Maryland; Laboratory of Vascular Thrombosis and Inflammation, National Heart, Lung, and Blood Institute, NIH, Bethesda, MD, USA, Bethesda, Maryland; Laboratory of Vascular Thrombosis and Inflammation, National Heart, Lung, and Blood Institute, NIH, Bethesda, MD, USA, Bethesda, Maryland; Laboratory of Infectious Diseases, National Institute of Allergy and Infectious Diseases, National Institutes of Health (NIH), Bethesda, MD, USA, Bethesda, Maryland; Critical Care Medicine Department, National Institutes of Health Clinical Center, Bethesda, MD, USA, Bethesda, Maryland; Advanced Lung Disease and Lung Transplant Program, Inova Fairfax Hospital, Falls Church, VA, USA, Bethesda, Maryland; Laboratory of Transplantation Immunotherapy, National Heart, Lung, and Blood Institute, NIH, Bethesda, MD, USA, Bethesda, Maryland; Laboratory of Transplantation Immunotherapy, National Heart, Lung, and Blood Institute, NIH, Bethesda, MD, USA, Bethesda, Maryland; Laboratory of Transplantation Immunotherapy, National Heart, Lung, and Blood Institute, NIH, Bethesda, MD, USA, Bethesda, Maryland; National Institutes of Health, Critical Care Medicine Department, Bethesda, Maryland; Critical Care Medicine, National Institutes of Health Clinical Center, Bethesda, Maryland

## Abstract

**Background:**

COVID-19 disease severity and outcomes have been linked to high antibody titers and a dysregulated neutrophil immune response. Here we query associations and connections between the endogenous SARS-CoV-2 antibody response and neutrophil activation in COVID-19.

**Methods:**

Baseline serum or plasma samples from 57 patients hospitalized on oxygen with COVID-19 were used to perform; 1) quantitative measurements of SARS-CoV-2 specific antibodies using a luciferase-based immunoprecipitation system assay, 2) quantitative measurements of neutrophil specific biomarkers using Luminex technology, and 3) neutrophil extracellular traps (NETs) as measured by myeloperoxidase-DNA (MPO-DNA) complexes by ELISA. Absolute neutrophil count (ANC) and immature granulocyte count (IGC) were measured from complete blood counts (CBC). Antibody levels were compared by disease severity using Wilcoxon rank-sum test and correlations were generated between antibody levels and neutrophil activation markers using Spearman’s correlation (SC).

**Results:**

In a cohort of hospitalized patients, severe/critical COVID-19 was associated with higher levels of nucleocapsid-IgA (p=0.011) as well as spike-IgG (p= 0.0007) compared to moderate disease, while spike-IgA and nucleocapsid-IgG showed similar associations, trending towards significance (Figure 1A). Levels of IgG-spike and IgG-nucleocapsid both had significant correlations with the ANC (SC 0.33, p = 0.029; SC 0.38 p = 0.012). All four antibody titers showed strong correlations with IGC, lactoferrin and lipocalin-2, evidence of emergency granulopoiesis. Further, S100A9, a component calprotectin correlated with spike-IgG and nucleocapsid-IgA levels (SC 0.29, p = 0.030, SC 0.29 p = 0.029). Lastly, we found circulating NETs correlated with spike IgA levels (SC 0.38 p = 0.006), and its correlations with IgG-spike and IgA-nucleocapsid additionally approached significance with NETs levels as well (Figure 1B).

Antibody Levels Correlate with Disease Severity and Neutrophil Activation Markers

**Figure:**
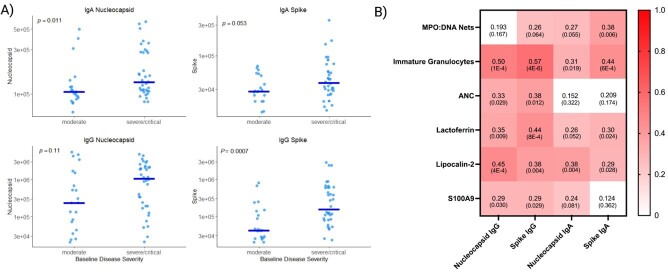

**Figure 1:** A) Levels of anti-Spike and anti-Nucleocapsid IgA and IgG levels measured in the serum of 57 unvaccinated hospitalized COVID-19 patients. Moderate illness represents ordinal scale 5 requiring low flow oxygen, while severe/critical patients represent ordinal scale 6 and 7, requiring high flow oxygen, non-invasive or mechanical ventilation, respectively. P values are compared by a Wilcoxon ranked sum test. B) Heatmap showing Spearman correlations between levels of anti-Spike and anti-Nucleocapsid IgA and IgG and markers of neutrophil activation. P values for individual correlations are represented in parentheses. MPO (myeloperoxidase), ANC (absolute neutrophil count), S100A9 (S100 calcium binding protein A9).

**Conclusion:**

Higher anti-spike and anti-nucleocapsid IgG and IgA levels associate with more severe COVID-19 illness. Further, endogenous SARS-CoV-2 specific antibody levels associate with markers of emergency granulopoiesis and neutrophil activation. Inhibiting antibody mediated neutrophil activation may improve outcomes in COVID-19.

**Disclosures:**

**All Authors**: No reported disclosures.

